# Light vs. Heavy Gold Foil and Crystal Gold

**Published:** 1870-12

**Authors:** Frank Abbott


### ARTICLE VII.

### *Light vs. Heavy Gold Foil and Crystal Gold.*

By Frank Abbott, D. D. S.

Read before the American Dental Association.

In nearly every journal devoted to the interest of the Dental profession, in nearly every Dental society, and in the private conversations of Dentists with each other, the subject of the different preparations of gold for filling teeth is referred to, and the supposed merits of each canvassed, in some instances to an enthusiastic extent. This has been more particularly the case recently or since the introduction of "heavy foils."

There was a time, probably in the recollection of some of our oldest practitioners, when it was impossible to obtain any but heavy gold; in fact, I have heard Dr. Eleazer Parmly state that during the first years of his practice he manufactured or prepared his own gold for filling teeth, which was done entirely with the rolling mill. At that time that was the best preparation of gold known to dentists. It has since been the constant desire of many dentists to obtain lighter foil. Some have even gone so far as to require it as light as $1\frac{1}{2}$ grs. to the sheet. Recently, however, the views and practice of many have changed, and now they require gold the same sized sheets, weighing from 10 to 480 grains. If you ask those gentlemen what advantages they derive from the use of these preparations, they will tell you that they can "work more rapidly," "make more perfect fillings," that they "can pack more gold into a cavity of the same size than they can of light or soft foil," and in short "it is the material with which perfection in filling teeth is arrived at.

Other gentlemen in equally as good standing in the profession will say that perfect plugs can not be made with such heavy materials, but claim that with crystal gold the greatest degree of perfecton is obtained. They will tell you that they can pack one quarter more of this gold into a given sized cavity than they can of any other preparation. And if any doubts are expressed in regard to the truth of the statements of these gentlemen, you are immediately written down an old fogy, a non-progressionest, or some other equally as interesting appellation. Now there is a class of Dentists who doubt very much the truth of these assertions; a class too, who are fond of advancement and do as much to advance the profession as any connected with it. This class is represented by those who believe their time and energies are spent to better advantage in using materials and teaching young and inexperienced men in the profession how to use materials, with which it is well known teeth can be saved, than in advocating the use of that which many believe tends to destroy untold numbers of teeth which might with some substance less difficult to adapt to the irregularities of cavities, have been saved. Imbibing to some extent the views of this latter class, and feeling that it was due as well to the profession as to the public, that a correct solution of the question of the best preparation of gold for filling teeth should be made, I have carefully conducted a series of experiments in the following manner and with the following results:

I first had several cavities prepared in steel of precisely the same size, to resemble nearly as possible cavities in teeth. In order to accomplish this more perfectly, several fine cuts were made in each with a graver, to represent the cuts of excavators, at the same time they are so arranged that the entire mass of each plug could be removed without marking its surface. This done, gold of the following numbers was then prepared (taking care that the pieces should all be as nearly the same weight as possible), viz: Nos. 2, 3, 4, 5, 6, 10, 20, 30, 40, 60, 120, 240, adhesive foil, and Nos. 3, 4, 5, and 6, soft foil, and Watts' crystal gold, No. 1.

An automatic mallet, with the same weight of stroke, and the same point, was used for each operation. The time occupied in condensing each was as follows:

| | | | | | | | |
|---|---|---|---|---|---|---|---|
| No. 2, | adhesive | ..........14 | minutes. | No. 40, | adhesive | ........ 28 | minutes. |
| " 3, | " | ..........14 | " | " 60, | " | .........31 | " |
| " 4, | " | ..........14 | " | " 120. | " | .........35 | " |
| " 5, | " | ..........16 | " | " 240, | " | .........36 | " |
| " 6, | " | ..........17 | " | " 3, | s'ft or non-adh. | 11½ | " |
| " 10, | " | ..........18 | " | " 4, | " " | 12 | " |
| " 20, | " | ..........21 | " | " 5, | " " | 14 | " |
| " 30, | " | ..........24 | " | " 6. | " " | 16½ | " |
| " 1, | Watt's crystal gold, 14 min. | | | | | | |

On removing the plugs, after their surfaces were finished, I found that Nos. 3. 4. 5. and 6, non-adhesive foil and the crystal gold plugs all presented the appearance of having been perfectly adapted to the cavities in every particular, while all the other cavities were more or less imperfect, even the very light Nos. 2, 3 and 4 presented innumerable minute openings into the body of the plug, and the fine cuts made by the graver were not perfectly filled, and as the gold increased in thickness, the imperfections increased correspondingly, so that from No. 10 to No 240 there was hardly an approximation to adaptibility. The cuts of the graver were not filled; every point where the gold was folded in the least an open space was left; in short they presented an appearance which might very appropriately be called the height of *imperfection*.

The weight of each plug was as follows:

| | | | | | | | |
|---|---|---|---|---|---|---|---|
| No. 2, | adhesive | ............15½ | grains. | No. 60, | adhesive | .........13½ | grains |
| " 3, | " | ............16 | " | " 120. | " | .........15 | " |
| " 4, | " | ............15½ | " | " 240, | " | ........ 15 | " |
| " 5, | " | ............16 | " | " 3, | non-adhesive | ...17 | " |
| " 6, | " | ............16 | " | " 4, | " | .........17 | " |
| " 10, | " | ............14½ | " | " 5, | " | .........17¼ | " |
| " 20, | " | ............14 | " | " 6, | " | .........17 | " |
| " 30, | " | ............15 | " | " 1, | crystal gold | .....14½ | " |
| " 40, | " | ............15 | " | | | | |

With the above facts before us, what becomes of the assertions that with heavy gold "time is saved"; "that more perfect operations are made," and "that more in weight can be packed into a cavity than can be of soft or non-adhesive gold of light Nos.? These questions I leave for the advocates of heavy and crystal gold to answer. In summing up this matter it seems unnecessary to add that the light Nos. of soft foil present decided advantages over any other material experimented with.

1st. It requires about one third the time under the same circumstances to manipulate them.

2d. Cavities are apparrently perfectly sealed with them, while all the others except crystal gold, present more or less imperfections; and

3d. From fifteen to eighteen per cent. more gold in weight is with the same pressure packed into a cavity.

